# Effects of Dantrolene Therapy on Disease Phenotype in Dystrophin Deficient mdx Mice

**DOI:** 10.1371/currents.md.e246cf493a7edb1669f42fb735936b46

**Published:** 2013-11-08

**Authors:** James L Quinn, Tony Huynh, Kitipong Uaesoontrachoon, Kathleen Tatem, Aditi Phadke, Jack H Van der Meulen, Qing Yu, Kannaboyina Nagaraju

**Affiliations:** Research Center for Genetic Medicine, Children’s National Medical Center, Washington, DC, USA; The Canberra Hospital, Endocrine Research Unit and Australian National University Medical School, Department of Paediatrics & Child Health, Canberra, Australia; Research Center for Genetic Medicine, Children’s National Medical Center, Washington, DC, USA; Research Center for Genetic Medicine, Children’s National Medical Center, Washington, DC, USA; Research Center for Genetic Medicine, Children’s National Medical Center, Washington, DC, USA; Research Center for Genetic Medicine, Children’s National Medical Center, Washington, DC, USA; Research Center for Genetic Medicine, Children’s National Medical Center, Washington, DC, USA; Department of Integrative Systems Biology, George Washington University School of Medicine and Health Sciences, Washington DC, USA; Research Center for Genetic Medicine, Children’s National Medical Center, Washington, DC, USA

## Abstract

Dystrophin deficiency causes contraction-induced injury and damage to the muscle fiber, resulting in sustained increase in intracellular calcium levels, activation of calcium-dependent proteases and cell death. It is known that the Ryanodine receptor (RyR1) on the sarcoplasmic reticular (SR) membrane controls calcium release. Dantrolene, an FDA approved skeletal muscle relaxant, inhibits the release of calcium from the SR during excitation-contraction and suppresses uncontrolled calcium release by directly acting on the RyR complex to limit its activation. This study examines whether Dantrolene can reduce the disease phenotype in the mdx mouse model of muscular dystrophy. We treated mdx mice (4 weeks old) with daily intraperitoneal injections of 40mg/kg of Dantrolene for 6 weeks and measured functional (grip strength, in vitro force contractions), behavioral (open field digiscan), imagining (optical imaging for inflammation), histological (H&E), and molecular (protein and RNA) endpoints in a blinded fashion. We found that treatment with Dantrolene resulted in decreased grip strength and open field behavioral activity in mdx mice. There was no significant difference in inflammation either by optical imaging analysis of cathepsin activity or histological (H&E) analysis. In vitro force contraction measures showed no changes in EDL muscle-specific force, lengthening-contraction force deficit, or fatigue resistance. We found Dantrolene treatment significantly reduces serum CK levels. Further, Dantrolene-treated mice showed decreased SERCA1 but not RyR1 expression in skeletal muscle. These results suggest that Dantrolene treatment alone has no significant beneficial effects at the tested doses in young mdx mice.

## Introduction

Duchenne muscular dystrophy (DMD) is an X-linked genetic disorder that affects 1 in 3500 boys in all populations 1. It is caused by a mutation in the dystrophin gene, leading to the absence of the cytoskeletal protein dystrophin 2. This protein connects the cytoskeleton of muscle fibers to the extracellular matrix. Without this anchoring protein, the muscle’s structure is compromised which in turn results in contraction-induced injury. This repeated injury leads to cycles of inflammation and muscle degeneration, which in turn leads to muscle weakening and loss of function. The repeated damage to the muscle fiber due to contraction also results in transient tears in the sarcolemma of dystrophic muscles, thereby allowing the influx of extracellular calcium 3. This elevated influx of calcium results in a sustained increase in intracellular calcium levels.

One of the major consequences of this calcium increase is the increased activation of calcium-dependent proteases that lead to protein degradation and eventually cell death 4. This increased degeneration from calcium-induced necrosis worsens the overall phenotype of DMD and hastens the loss of ambulation and eventual death due to respiratory and cardiac complications. Therefore, targeting mechanisms that control calcium should have therapeutic benefits. In fact, restoring calcium homeostasis by increasing calcium channels or the use of drugs that increase cellular proteins that stabilize calcium channels has been shown to improve muscle function and strength 5 ^,^ 6 ^,^ 7.

In skeletal muscle, contraction is induced through a process of calcium-induced calcium release. This occurs when the Ryanodine receptor (RyR1) on the sarcoplasmic reticular (SR) membrane interacts with voltage-dependent Ca^2+^ channels on the T-tubules such as dyhydropyridine receptors (DHPRs) and allows calcium release from the SR into the sarcoplasm when activated 8. This complex is activated by signals proliferated along the T-tubules and leads to muscle contraction.

Another channel of interest for calcium handling within muscle fibers is the sarco/endoplasmic reticulum Ca^2+^-ATPase (SERCA1), a calcium ATPase found on the SR membrane in muscle fibers. This protein is responsible for pumping calcium out of the sarcoplasm and back into the SR following excitation 9. It has been demonstrated that this protein is dysfunctional in mdx mice and further contributes to increased levels of sarcoplasmic calcium 10.

Dantrolene Sodium is an FDA approved skeletal muscle relaxant that has been safely administered to DMD patients without harmful side effects 11. Dantrolene binds to the RyR1 receptor and inhibits the release of calcium from the SR during excitation-contraction by blocking its activation 12.

We will use Dantrolene treatment to decrease intracellular calcium levels and thus decrease the amount of calcium-induced necrosis and improve the overall phenotype of the treated mice. To test the efficacy of this treatment regime, we have performed a pre-clinical trial in mdx mice and will evaluate improvement using several criteria: functional, behavioral, histological, and molecular assays.

## Methods

**Animal and Drug Dosing Regimen:**

Wild type (C57BL/10ScSn/J) and mdx (C57BL/10ScSn-mdx/J) mice were obtained from Jackson Laboratory (Bar Harbor, ME) and bred in house in our Animal Facility. All mice were acclimated for four weeks before testing. All handling and experimentation with mice was conducted in accordance with our IACUC guidelines under approved protocols. Mdx mice were assigned to treatment groups randomly based on bodyweight (BW). Beginning at 4 weeks of age, all animals received daily (7 day/week) single intraperitoneal injections of water or Dantrolene at a concentration of 40mg/kg for 6 weeks. Functional testing was conducted at 8 weeks of age followed by optical imaging the next week. At 10 weeks of age the mice were sacrificed and tissue samples were immediately collected, weighed, snap frozen in liquid nitrogen-cooled isopentane, and stored at -80˚C for later processing.

**Grip Strength Measurement:**

After four weeks of treatment, forelimb and hindlimb GSMs were performed as previously described 13 but with an increase in the acclimation period from three to five consecutive days. The GSMs were collected in the morning hours over a 5-day period, with maximum values for each day over this period averaged to obtain absolute GSM values (Kgf) or normalized to BW (recorded on the first day of testing) for normalized GSM values (Kgf/kg).

**Open-field Behavioral Activity Measurements (Digiscan):**

After four weeks of treatment, voluntary locomotor activity was measured using an open-field digiscan apparatus (Omnitech Electronics, Columbus, Ohio) as previously described 13 except that the mice were acclimated for four days before data was collected. Following acclimation, data was recorded over a 1-hour period each day for four consecutive days. Acclimation and collection of data was performed in the morning hours for all mice.

***In vitro*** **Force Contraction:**

Prior to sacrifice, the extensor digitorium longus (EDL) muscles of the right hindlimbs were removed for comparison of *in vitro* force contractions between groups as previously described 14. Briefly, the distal tendon of the muscle was tied securely to the lever arm of a servomotor/force transducer (model 305B) (Aurora Scientific, Aurora, Ontario, Canada) and the proximal tendon was fixed to a stationary post in the bath. The muscles were stimulated between two stainless steel plate electrodes. At optimal muscle length, the force developed was measured during trains of stimulation (300 milliseconds) with increasing frequencies until the highest plateau was achieved. The force generated to obtain the highest plateau was used to determine specific force and is expressed as mN/mm^2^. Additionally, the muscle was subjected to a protocol of ten lengthening contractions separated by one minute rest intervals. The muscle was stimulated at 250Hz for 300ms allowing the muscle to generate force immediately followed by a lengthening over 10% of muscle length at a velocity of two fiber lengths/second. Finally, the muscle was subjected to a fatigue protocol consisting of 60 isometric contractions for 300 milliseconds each, once every 5 seconds. The frequency at which the EDL muscles were stimulated is 250Hz. The force was recorded every minute during the repetitive contractions and again five minutes after to measure recovery.

**Live Animal Optical Imaging:**

After five weeks of treatment, quantification of inflammation in forelimb and hindlimb muscles of mice was achieved using live-animal optical imaging of Cathepsin-B (CTSB) enzyme activity using a caged near-infrared substrate (ProSense 680) as previously described 15. Briefly, mice were injected intraperitoneally with 1.5 nmol of ProSense 680 (VisEn Medical). Areas of scanning were defined at 1.0-mm resolution. Cathepsin activity was measured from forelimb and hindlimb muscles and defined as intensity in terms of photon counts/mm^2^ using the Optiview 2.0 software Optix.

**Serum creatine kinase (CK) measurements:**

The blood was collected by cardiac puncture immediately after euthanasia by carbon dioxide inhalation. Blood was spun down and serum collected. Creatine kinase levels (U/L) were analyzed using a standard spectrophotometric method with enzyme-coupled assay reagent, CK10 (Thermo Fisher Scientific, Waltham, MA), according to the manufacturer’s instructions 16.

**Histopathology Scoring:**

Directly following euthanasia, the tibialis anterior (TA) muscles were removed and preserved in formalin. For histological analysis, muscles were embedded into paraffin blocks and subsequently sectioned and stained with haematoxylin and eosin (H&E) by Histoserv (Germantown, MD). The slides were blinded and randomized and five digital images at 20X magnification were taken of each using an Eclipse E800 (Nikon, Japan) microscope, and each was assessed for the nine following criteria; fibers/field, central nuclei/fiber, peripheral nuclei/fiber, total nuclei/fiber, fibers with central nuclei/field, ratio of central to peripheral nuclei/field, regenerating fibers/field, percentage of central nucleated fibers and inflammatory foci using Image J (NIH) as previously reported 13.

**Western blotting:**

SERCA1 ATPase protein levels were measured in Gastrocnemius muscles from the mdx treatment and control groups. Frozen muscle sections were homogenized as previously described 17 and equal amounts (50ug) were loaded and run in a reducing SDS-PAGE gel (Invitrogen, Grand Island, NY) at 150V. The gel was then blotted transferred onto PVDF membranes and incubated overnight with anti-SERCA1 ATPase monoclonal antibody (1:2500, Thermo Scientific, Waltham, MA) at 4°C. After washing, membranes were probed with HRP-conjugated anti-mouse IgG1 (1:2000, Invitrogen) and incubated with ECL Western Blotting Substrate (GE Healthcare, Piscataway, NJ) and processed on X-ray film (Denville Scientific, South Plainfield, NJ). Densitometry analysis was performed and normalized to β-actin (1:1000, Santa Cruz).

**Quantitative PCR (qPCR):**

RNA was isolated from frozen Gastrocnemius muscle using an integrated Trizol and RNeasy minikit (Qiagen, Valencia, CA) protocol with DNase I (Qiagen) treatment. cDNA was obtained using Reverse Transcription System (Promega, Madison, WI). The Taqman Gene Expression Assays Hprt (Mm01545399_m1) and Ryr1 (Mm01175211_m1) were used for analysis. All PCR reactions were carried out using Taqman Gene Expression Master Mix on the 7900HT Fast Real-Time PCR System under the following thermal cycle conditions: initial steps (UNG activation - 50°C for two minutes; DNA Polymerase activation - 95°C for 10 minutes) and 40 cycles with a melting temperature of 95°C for 15 seconds and an annealing/extend temperature of 60°C for 60 seconds.

**Statistical Analysis:**

For all proposed experiments and procedures, one-way ANOVA with Dunnett’s post-test comparison against a control was used for experiments involving more than two groups. A two-tailed Student’s t test was used for experiments involving only two groups. Significance was defined as a p-value less than 0.05. All data is expressed as mean ± standard error of the mean (S.E.M.). Prism statistical software (GraphPad, La Jolla, CA) was used for statistical analyzes. qPCR results were analyzed for significant differences by a 2000-sample pairwise fixed reallocation randomization test using REST software 18.

## Results

**Effect on body weight**

****Bodyweight of each mouse was measured at four weeks of age and recorded every week of treatment up until sacrifice at ten weeks of age. All three groups weighed approximately the same at the beginning of treatment with BL10 mice averaging 6.47% weight increase each week of treatment while untreated and treated mdx mice averaged 10.33% and 10.53% respectively. This resulted in a significant difference (P-value = 0.0115) between the bodyweights of the BL10 group and the mdx groups starting at two weeks of treatment and continuing until the end of the trial. There was no significant difference in bodyweight over time between the two mdx groups (Fig. 1).

**Body weight measurements during treatment:**
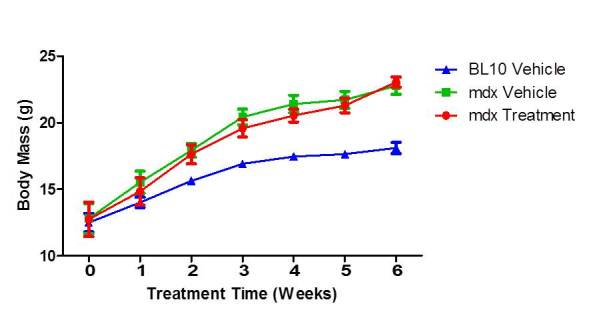
Body weight was recorded each week over the entire duration of treatment. The two mdx treatment groups showed no significant change in body weight of the course of treatment after the mdx mice were broken up into groups based on normalizing bodyweight. N=8

**Effect on functional and behavioral assays**

****Grip strength and open field testing for mdx and BL10 mice was performed after 4 weeks of treatment. Vehicle-treated mdx mice showed a reduction in absolute forelimb strength (7.88%) compared to BL10 mice. Treatment of mdx mice with Dantrolene resulted in a 16.69% reduction in strength compared to the untreated mdx mice (Fig. 2A). Because we found significant differences in bodyweight between BL10 and mdx mice, we also normalized strength to body weight. Untreated mdx mice showed a significant decrease (24.23%) in normalized forelimb strength relative to the BL10 mice. Mdx mice treated with Dantrolene showed a 13.58% decrease compared to the untreated mdx group (Fig. 2B). Hindlimb measurements were unable to ascertain a difference between mdx and BL10 mice even after normalization (data not shown).

**GSM results of wild type and mdx mice:**
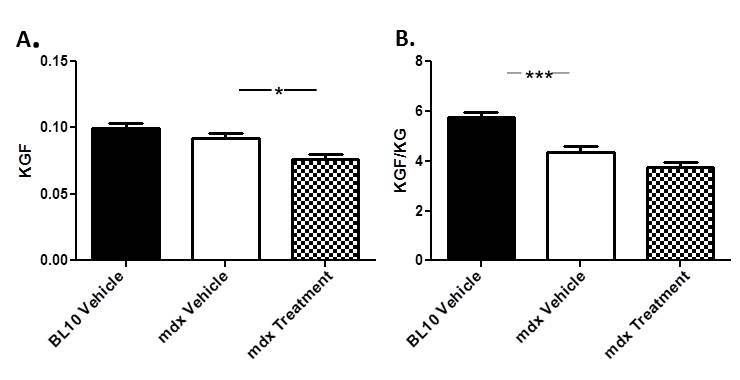
Grip strength measurements were performed at 4 weeks of treatment. A) Absolute Forelimb B)Forelimb normalized to body weight. * P ≤ 0.05, *** P ≤ 0.001. N=8

Activity was measured using open field activity chambers. There was no significant change in any measured parameters, including horizontal and vertical activity as well as movement and rest time, between any of the groups (Table 1).

**Table 1: Open field activity measured parameters:** Comparisons between BL10 and mdx Vehicle-treated mice as well as both mdx mice groups yielded no significant differences. N=8

Measurement	BL10 Vehicle	mdx Vehicle	mdx Treatment
Horizontal Activity (arbitrary units)	1785.7 ± 81.2	1463.0 ± 99.9	1259.2 ± 118.7
Vertical Activity (arbitrary units)	26.4 ± 5.8	87.8 ± 25.7	41.4 ± 12.2
Total Movement Time (seconds)	71.9 ± 8.5	64.0 ± 8.1	53.0 ± 8.4
Total Rest Time (seconds)	528.0 ± 8.5	536.0 ± 8.0	547.0 ± 8.4

**Effect on muscle inflammation**

****We have previously shown that cathepsin activity in fore and hindlimbs is significantly increased in mdx mice 20. We assessed cathepsin activity using optical imaging after 5 weeks of treatment. Untreated mdx mice showed a significant (84.87%) increase in cathepsin activity in the forelimbs using optical imaging compared to BL10 mice. They also showed a 42.41% increase in hindlimb cathepsin activity. Mice treated with Dantrolene showed no significant decrease (18.17%) in inflammation in the forelimbs compared to untreated mdx mice. Alternatively, no change was found in the hindlimbs (Fig. 3).

**Analysis of muscle inflammation using optical imaging of cathepsin:**
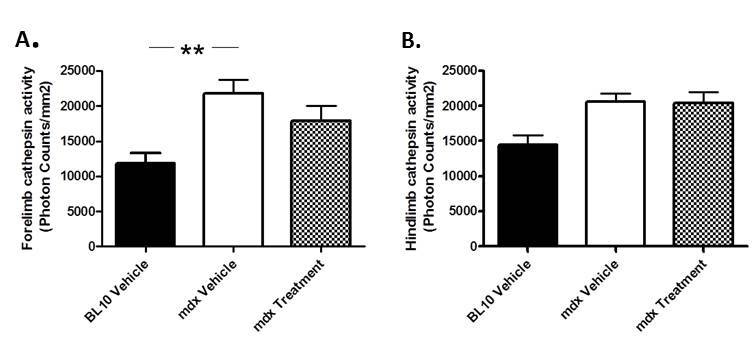
Cathepsin activity was measured from A) forelimb and B) hindlimb muscles as a quantification of inflammation. Activity is defined in terms of photon counts/mm2. ** P ≤ 0.01

**Effect on measured histological parameters**

****Hematoxylin and eosin (H&E) stained sections from the TA of each animal were analyzed in blinded fashion. Dantrolene treatment did not significantly alter any of these parameters (fibers/field, central nuclei/fiber, peripheral nuclei/fiber, total nuclei/fiber, fibers with central nuclei/field, ratio of central to peripheral nuclei/field, regenerating fibers/field, percentage of central nucleated fibers and inflammatory foci) compared to the vehicle-treated mdx mice (Table 2).

**Table 2: Comparison of histological parameters in TA muscle:** Analysis of muscle histology performed on TA muscle of each animal in a blinded fashion. Comparisons of ten standard parameters yielded no significant change between the two mdx groups. N=8

Measurement	mdx Vehicle	mdx Treatment	P-value
Fibers/Field	179.45 ± 4.57	191.47 ± 8.93	0.2675
Central Nuclei/Fiber	0.713 ± 0.03	0.693 ± 0.03	0.6490
Peripheral Nuclei/Fiber	0.717 ± 0.03	0.702 ± 0.03	0.7311
Total Nuclei/Fiber	1.430 ± 0.06	1.396 ± 0.05	0.6541
Fibers with Central Nuclei/Field	98.125 ± 3.28	104.133 ± 4.93	0.3392
Ratio of Central to Peripheral Nuclei/Field	0.00559 ± 0.0003	0.00527 ± 0.0003	0.4533
Regenerating Fibers/Field	2.625 ± 0.78	3.844 ± 0.98	0.3544
% of Central Nucleated Fibers	71.28% ± 3.33	69.33% ± 2.63	0.6489
Inflammation Foci/Field	0.675 ± 0.12	1.133 ± 0.51	0.4073

**Effect on *in vitro* muscle force, fatigue resistance, and lengthening contractions**

**** *In vitro* force analysis was performed on the EDL muscle of each mouse prior to sacrifice. The EDL muscles of mdx mice had a significant decrease (31.98%) in specific force relative to BL10 mice. Dantrolene, however, did not significantly improve it (Fig. 4A). Additionally, mdx mice were significantly less resistant to damage occurring from lengthening compared to BL10 mice (Fig. 4B). Treatment with Dantrolene showed no improvement in muscle force generated after lengthening contractions (Fig. 4B). Finally, no significant differences were observed between any of the groups in muscle fatigue or recovery (Fig. 4C).

**In vitro force assessment performed on the EDL muscle:**
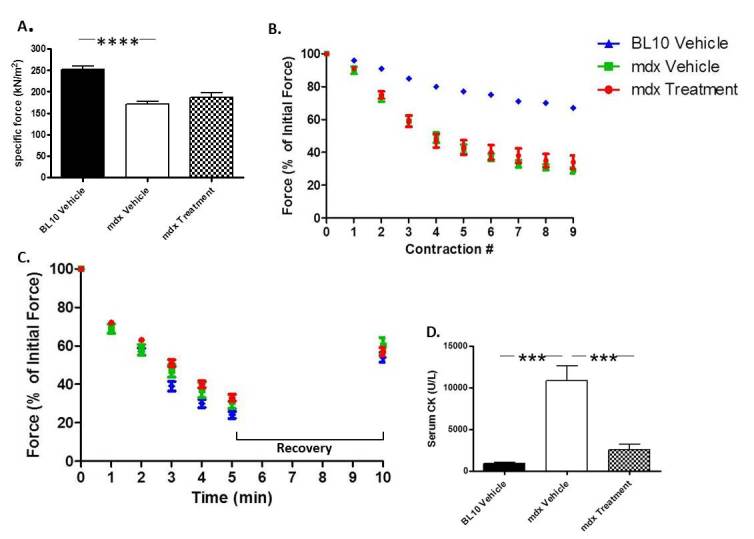
No significant difference was observed in any of these measurements including A) specific muscle force, B) lengthening contractions to measure how the muscle responds to damage from over lengthening, or C) measuring the muscle’s ability to resist and recover from fatigue. D) Serum was collected at sacrifice via cardiac puncture and CK levels were measured. *** P ≤ 0.001, **** P ≤ 0.0001. N=8

Effect on serum creatine kinase levels

****Serum collected at sacrifice was analyzed to assess creatine kinase levels. In untreated mdx mice, there was an 11.76 fold increase in creatine kinase in serum collected from mice prior to sacrifice relative to wild type controls. Treatment with Dantrolene resulted in a significant decrease (76.42%) in serum CK levels compared to untreated mdx mice (Fig. 4D).

**Effect on SERCA1 protein levels in muscle**

****To evaluate whether Dantrolene treatment altered SERCA1 expression, we did western blotting and densitometry normalized to β-actin. This was performed on protein extracted from frozen gastrocnemius muscle. Dantrolene-treated mice showed a significant decrease (33%) in SERCA1 levels compared to vehicle-treated mdx mice (Fig. 5).

**Western blot and densitometry analysis of SERCA1:**
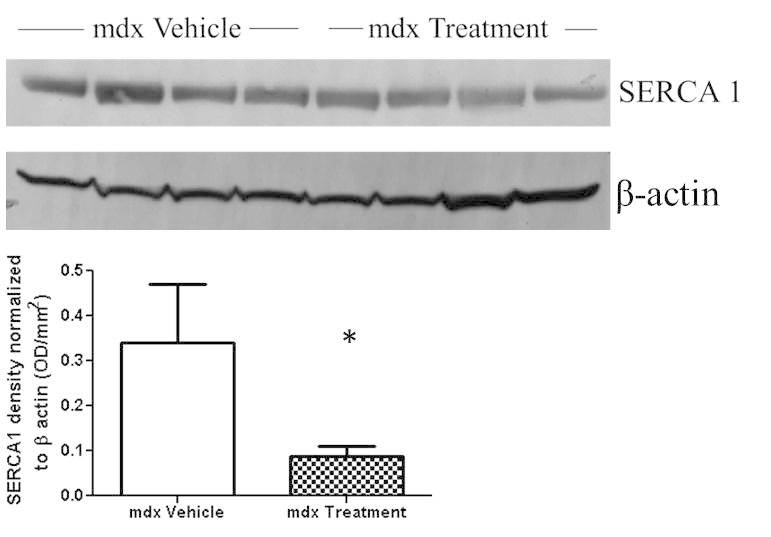
Western blot and densitometry analysis performed on protein extracted from gastrocnemius muscle to measure SERCA1 protein levels in skeletal muscle. * P ≤ 0.05. N=4

**Effect on RyR1 gene expression levels in muscle**

In order to determine the effect of Dantrolene treatment on Ryr1 in skeletal muscle, qPCR to measure RyR1 levels was performed. Ryr1 gene expression was normalized to HPRT. We did not find differences in RyR1 expression between the two mdx mouse groups (data not shown).

## Discussion

In this study we demonstrated that Dantrolene treatment has no significant beneficial effects at the tested dose in young mdx mice. Dantrolene failed to improve the muscle phenotype as measured by functional, behavioral, histological, and imaging assays. The exception being CK levels, which were significantly decreased by Dantrolene treatment.

Dantrolene is a known muscle relaxant and used for the treatment of malignant hyperthermia 11. It decreases the calcium released in skeletal muscle upon stimulation, resulting in decreased muscle contraction 19. In fact, functional assessments such as grip strength and open field activity were decreased in Dantrolene-treated mice compared to their vehicle-treated counterparts. This is likely a result of the muscle relaxant properties of the drug. This further indicates that the treatment does not improve muscle function or strength. To minimize variation in the functional and behavioral assays, only female mice were used because there are clear gender differences in mdx muscle pathology between sexes 21 and to reduce variability others and we have suggested that pre-clinical tests should be performed on groups of mdx mice of the same sex 22.

It is clear from the results that Dantrolene did not reduce inflammation in mdx mice. Cathepsin B activity (macrophage/muscle) as measured by optical imaging is unaltered in the two mdx mouse groups. These findings were confirmed by analysis of H&E sections that also indicate no change in the histological profile, including inflammation, between the two groups.

Even though it has been shown previously that RyR1 is leaky in dystrophic muscle and contributes to the disease phenotype, and drugs that stabilize RyR1 are known to be effective in muscular dystrophy 23, we could not see a similar effect with Dantrolene in this experiment. The reasons for this discrepancy are not clear, but could be due to the muscle relaxant properties of the drug.

Dantrolene has previously been shown to enhance exon skipping in mdx mice but not improve muscle strength on its own 24. Our study confirms their findings despite using a higher dose of the drug to treat older mdx mice for a longer period of time (8 weeks). In addition, we conducted additional functional assays to determine the effect of the drug.

Lack of efficacy in our study could be a result of beginning treatment too late in the disease progression, at a point where significant damage had already occurred in the muscle fibers. Previous studies have demonstrated that calpains, calcium-dependent cytosine proteases, are present at elevated levels in mdx muscle by 4 weeks of age 25. This means that calcium-induced necrosis had already initiated by the time we started treatment. It is unlikely that improved calcium handling in previously damaged fibers would reverse the damage, but only slow further deterioration. Additionally, it is quite possible that a longer duration of treatment is necessary to observe any benefit from the drug in mdx mice.

Our analysis of the serum CK levels did show a significant decrease in the amount of CK in the blood. This trend is consistent with previously shown data 26. Based on the histology and functional assays, it is unlikely that this change is based on improved muscle health. Instead, it is highly likely that this decrease in serum CK is a result of decreased activity. As a muscle relaxant, Dantrolene slowed down the breathing rate and reduced activity in the mice 19, reducing CK levels in the blood.

To determine whether Dantrolene target, RyR1, was engaged, we evaluated a sarco/endoplasmic reticulum Ca^2+^-ATPase (SERCA1). This calcium ATPase is found on the SR membrane in muscle fibers, and is responsible for pumping calcium out of the sarcoplasm and back into the SR. We expected that Dantrolene treatment and the resulting intracellular calcium decrease would result in an increase in SERCA1 levels in muscle fibers based on previous research with rat hearts 27. We performed Western blotting on gastrocnemius muscles and found a 33% decrease in SERCA1 protein levels compared to mdx vehicle-treated mice. We suspect this difference in effect is likely due to the different muscle type, dosage, duration, and model. This assay however, establishes that Dantrolene did affect calcium handling within mdx muscle fibers but it did not result in an improved disease phenotype.

Additional assays that we performed on isolated EDL muscles indicate that not only strength, but also muscle recovery during *in vitro *fatigue and lengthening contraction protocols demonstrated no effect. Additionally, the histological scoring showed no significant change in any of the eight measured parameters, indicating existing damage of the fibers was not altered. Our data suggests that during early necrosis when there is cycles of damage, regeneration, and re-injury; administration of Dantrolene is ineffective. Therefore, we conclude that Dantrolene alone at this dose does not improve the overall phenotype of the mdx mouse.

## Correspondence

Corresponding author:

Kanneboyina Nagaraju, DVM, Ph.D.

Research Center for Genetic Medicine

Children’s National Medical Center

111 Michigan Ave. NW

Washington DC 20010

Email: knagaraju@cnmcresearch.org

Phone (202)476-6220

## Competing Interest Statement

The authors have declared that no competing interests exist.
